# Extraordinary slow degradation of dissolved organic carbon (DOC) in a cold marginal sea

**DOI:** 10.1038/srep13808

**Published:** 2015-09-08

**Authors:** Tae-Hoon Kim, Guebuem Kim, Shin-Ah Lee, Thorsten Dittmar

**Affiliations:** 1 School of Earth and Environmental Sciences/RIO, Seoul National University, Seoul, 151-747, Republic of Korea; 2 Department of Earth and Marine Sciences, Jeju National University, Jeju, 690-756, Republic of Korea; 3 Research Group for Marine Geochemistry (ICBM-MPI Bridging Group), Institute for Chemistry and Biology of the Marine Environment (ICBM), Carl von Ossietzky University, 26111 Oldenburg, Germany, and Max Plank Institute for Marine Microbiology (MPI), Bremen, Germany

## Abstract

Dissolved organic carbon (DOC) is the largest organic carbon reservoir in the ocean, and the amount of carbon in this reservoir rivals that in atmospheric CO_2_. In general, DOC introduced into the deep ocean undergoes a significant degradation over a centennial time scale (i.e., ~50 μM to ~34 μM in the North Atlantic and Mediterranean Sea). However, we here show that high concentrations of DOC (58 ± 4 μM) are maintained almost constantly over 100 years in the entire deep East/Japan Sea (EJS). The degradation rate in this sea is estimated to be 0.04 μmol C kg^−1^ yr^−1^, which is 2–3 times lower than that in the North Atlantic and Mediterranean Sea. Since the source of DOC in the deep EJS is found to be of marine origin on the basis of δ^13^C-DOC signatures, this slow degradation rate seems to be due to low temperature (<1 ^o^C) in the entire deep water column. This observational result suggests that the storage capacity of DOC in the world ocean is very sensitive to global warming and slowdown of global deep-water overturning.

Most of the DOC in the ocean is produced by marine organisms and removed by abiotic processes, such as photochemical oxidation^1^,^2^ and microbial degradation^3^. DOC is a highly complex mixture of compounds^4^, each having a different reactivity. Although DOC plays an important role in global carbon cycling^5^,^6^,^7^, the mechanisms underlying the production and removal of DOC have yet to be clarified^4^. The objective of this study was to examine the degradation rate of DOC in a very cold deep ocean (EJS), which may infer the sensitivity of DOC storage capacity in warming oceans.

The EJS is a semi-enclosed marginal sea and is divided into three major basins (Japan Basin, Ulleung Basin, and Yamato Basin), all of which are deeper than 2000 m (Fig. 1). The deep water masses (at depths greater than 1000 m) in the EJS are formed by deep convection or brine rejection in the Japan Basin^8^,^9^,^10^,^11^. Using salinity and oxygen isotope budgets^9^, the rate of potential bottom-water formation due to brine rejection (at depths greater than 2500 m) was estimated to be approximately 4 × 10^12^ m^3^ yr^−1^. This brine rejection accounts for 25–35% of the subsurface water formed in the EJS. The turnover time of the EJS deep water mass was determined to be ~100 years by using radionuclides such as ^14^C and ^226^Ra^12^,^13^ by assuming steady-state conditions.

## Results and Discussion

**Figure 1:**
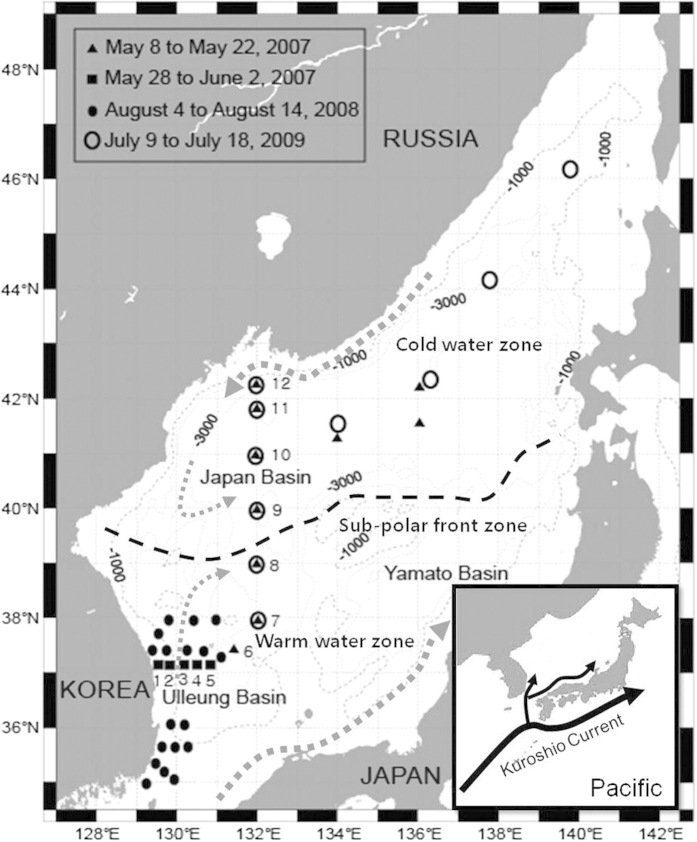
A map showing sampling stations for vertical dissolved organic carbon (DOC) profiles in the East/Japan Sea. Seawater samples for DOC analysis were collected with a Niskin sampler from 30 stations in the southern area and from 15 stations in the northern area of the EJS. The hydrological and biogeochemical surveys were conducted during four periods: May 8 to 22, 2007, on board the R/V M.A. Gagarinsky of the Pacific Oceanological Institute (POI), Russia; May 28 to June 2, 2007, onboard the R/V Tam-Yang of the Pukyung National University (PKNU), Korea; August 4 to 14, 2008, onboard the R/V HaeYang 2000 of the National Oceanographic Research Institute (NORI), Korea; and July 9 to 18, 2009, on board the R/V M.A. Lavrentyev of the POI, Russia. Map was created using Adobe Illustrator.

**Figure 2:**
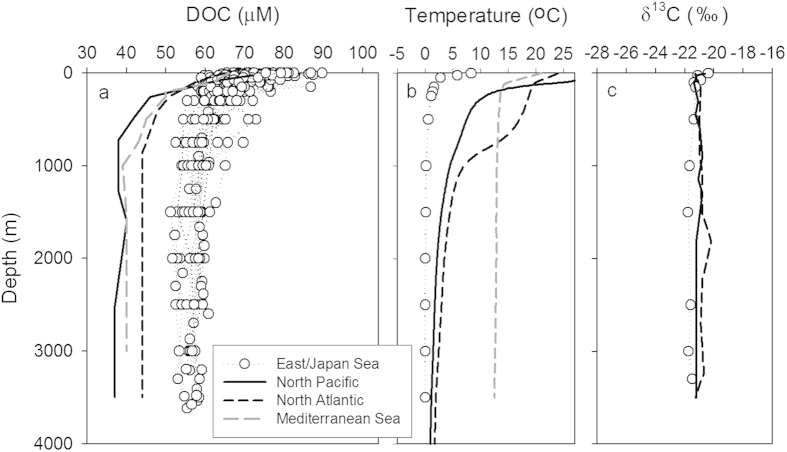
Vertical profiles of dissolved organic carbon (DOC), potential temperature, and δ^13^C-DOC. (**a–c)** Vertical profiles of (**a**) DOC, (**b**) potential temperature, and (**c**) δ^13^C-DOC in the East/Japan Sea (open circle), North Pacific (black lines), North Atlantic (black dotted lines), and Mediterranean Sea (gray dotted lines). Data in the North Pacific, North Atlantic, and Mediterranean Sea are from Hawaii Ocean Time-series (HOT), Bermuda Atlantic Time-series (BATS), and Santinelli *et al*. (ref. 26), respectively. Samples for δ^13^C-DOC in the East/Japan Sea were collected in station 10 (42 °N). The depth profiles of δ^13^C-DOC in the major oceans are from the North Central Pacific (31 °N) and the Sargasso Sea (31 °N).

Vertical profiles of DOC concentration were determined on several cruises covering the main EJS during the course of three years (Fig. 1). The average DOC concentrations in the surface layer (0–200 m) and in the deep (>200 m) layer over the entire area of the EJS were 68 ± 6 and 58 ± 4 μM, respectively (Fig. 2). In general, the DOC concentrations in the surface layer of EJS were comparable to those in the major world oceans (60–80 μM)^14^,^15^,^16^. However, the DOC concentrations in the deep layer of the EJS are significantly higher than those in the major oceans (34–43 μM)^6^,^17^,^18^ (Fig. 2) and slightly higher than or similar to those in the Arctic Ocean (54 ± 3 μM, up to ~4000 m)^19^ and the Nordic seas (50 μM, up to ~3500 m)^20^, where deep water formation occurs. In the Greenland Sea, one of the Nordic seas, the concentrations of DOC decrease from the surface (60 μM) to the deep layer (53 μM, <1500 m depth) and reach a constant DOC concentration (50 μM) in the deeper layer (>1500 m depth)^20^. Therefore, the concentration of DOC in the deep bottom water of the EJS seems to be the highest reported so far in the world oceans.

In the Arctic and Nordic Seas, the elevated concentrations of DOC are related to downwelling of waters that are enriched in terrigenous DOC^19^,^20^. However, there is no large river that directly drains into the EJS. In addition, export of DOC in the deep EJS to the Pacific Ocean can be ignored since the EJS is connected to the Pacific Ocean only through the sills which are shallower than 150 m. The δ^13^C-DOC in the EJS ranged from −20.4 to −21.7% (avg.: −21.3 ± 0.4%), similar to that in the North Pacific (avg.: −21.2 ± 0.2%) and in the North Atlantic (avg.: −21.0 ± 0.3%) oceans^21^ (Fig. 2). The enriched δ^13^C values in these oceans are different from terrigenous C_3_ plant material (−28 to −30%) indicating that the main fraction of the DOM is of marine origin (δ^13^C values of plankton organic matter = −18 to −20%). However, we cannot exclude the possibility that the Tsushima Current, which brings Yangtze River water into the EJS, may contribute some terrigenous DOC to the EJS. In addition, the DOC production from sinking particulate organic carbon (POC) could result in high DOC in the EJS. However, despite most of the sinking POC (>80%) being generally degraded between 200 and 1600 m^22^, the DOC profiles were uniform throughout the entire depths, down to 3500 m, indicating that DOC production in the deep ocean is not significant over the time scale of deep water mixing. The total export production (including sinking particles) through 200 m in the EJS has been estimated to be 99 Tg yr^−1^ (8.25 mol C m^−2^ yr^−1^) by using ^3^H-^3^He isotopes^22^, which in general is not unusually higher than in other oceans.

**Figure 3:**
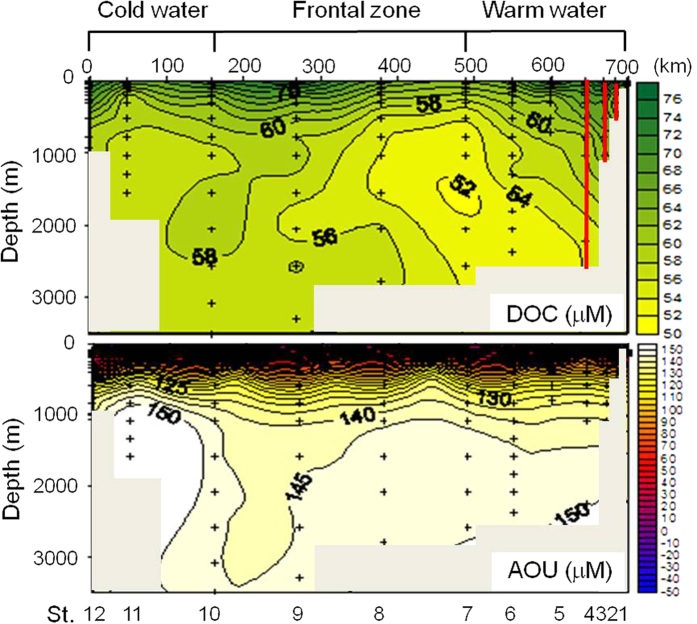
Transectional contours of dissolved organic carbon (DOC) and apparent oxygen utilization (AOU) in the East/Japan Sea. Distributions of DOC and AOU in the East/Japan Sea during May 8 to 22, 2007 and May 28 to June 2, 2007. Red lines indicate distributions of DOC reported by Kim and Kim (ref. 23).

The concentrations of DOC in the deep layer of the EJS in May 2007 decreased slightly from the north (59 ± 3 μM) to the south (55 ± 2 μM) along the deep-sea current flows, which was associated with biological degradation reflected in apparent oxygen utilization (AOU) (Fig. 3). Considering the fact that these DOC profiles include the entire East Sea through several cruises, including the southwestern EJS stations^23^, over the last 10 years, the DOC concentrations in the deep EJS were remarkably stable, and neither systematic nor significant differences were observed among the stations.

In order to determine the characteristics of DOC degradation in the EJS, it was necessary to compare DOC degradation rates for the same DOM quality and the same age following deep water formation. In this connection, the degradation rate of DOC in the deep EJS was compared with those of the Atlantic Ocean and the Mediterranean Sea, which have similar deep water formation modes (thermohaline circulation). Because the turnover times of the EJS and Mediterranean Sea are about 100 years, we used the DOC difference in the entire regions of these marginal seas. In the case of the Atlantic Ocean, we used the difference in DOC concentration between the Greenland Sea water and the water mass around 50 ^°^N, which was found to be approximately 100 years old based on ^14^C dating^24^.

The slopes of DOC concentrations over the time scale of 100 years were obtained from the highest (59, 50, and 48 μM) to the lowest (55, 42, and 34 μM) concentrations in the EJS, Atlantic Ocean^25^, and Mediterranean Sea^26^, respectively. A major fraction of the DOC mineralization was assumed to have occurred during downwelling. Then, the degradation rate of DOC was estimated to be 0.04, 0.08, and 0.14 μmol C kg^−1^ yr^−1^ in the EJS, Atlantic Ocean, and, Mediterranean Sea respectively. The degradation rate in the EJS is much lower than those of the Mediterranean Sea and the Atlantic Ocean.

In order to determine the contribution of DOC oxidation to oxygen consumption in deep EJS, DOC is plotted against AOU. The plotting was performed after converting AOU to carbon equivalents [AOU-C_eq_ (μM C) = AOU (μM O_2_) × 0.72]^16^. A ΔC/ΔO ratio of 0.72 was derived from the Redfield stoichiometry (C:O:N:P = 106:42:16:1)^27^. The slope (−0.13) of the linear regression of DOC concentration versus AOU-C_eq_ in the deep EJS indicates that oxidation of DOC is responsible for 13% of the oxygen utilization in the deep EJS. This value in the deep EJS is similar to that (14 ± 3%) in the North Atlantic Deep Water^25^, but much higher than that (approximately 32%) in the eastern Mediterranean Sea^28^.

The reason behind the low degradation rates remains speculative at this point, but it is most likely associated with the difference in water temperature. The water temperature in the EJS is **<**1 ^°^C (Fig. 2), which is lower than that in the Atlantic Ocean (3 ^°^C, range from 2.2–3.5 ^°^C) and in the Mediterranean Sea (~13 ^°^C, range from 12.8–13.7 ^°^C). Carlson *et al*. (ref. 25) suggested that the degradation rates of DOC derived from single end-member and multiple linear regression models decrease exponentially as temperature decreases although temperature cannot be the sole control of DOC degradation. DOM quality other than temperature alone might control DOM degradation rates^29^,^30^,^31^.

The water temperature increased by 0.1–0.5 ^°^C in the upper 1000 m and by 0.01 ^°^C below 2000 m from the 1950s to 1996 in the EJS because of the recent slowdown in deep-water formation^32^. Similarly, the rate of anthropogenic CO_2_ accumulation in the deep layer of the EJS decreased considerably from 1992 to 2007 owing to the considerable weakening of the overturning circulation^33^. It is unknown whether this recent small change has resulted in DOC reduction in the deep EJS during the last few decades. However, our results from EJS in comparison with other seas suggest that global carbon cycles could be significantly affected by the warming and slow-down of water overturning in marginal seas.

## Methods

The samples for DOC and δ^13^C-DOC were filtered onboard through a pre-combusted syringe glass-fiber filter (Whatman, 0.7 μm, 25 mm) into pre-combusted glass ampoules (550 °C for 5 h). The samples were then acidified to pH 2 with 6 M HCl and stored at 4 °C for preservation until further analysis. All sampling procedures were performed in a clean bench (class 100). The DOC concentrations were measured using a TOC-V_CPH_ analyzer (Shimadzu, Japan), as described elsewhere^34^. Briefly, the acidified seawater sample was bubbled with high-purity air gas (purity: 99.999) to completely purge inorganic carbon species in the injection system. Carrier gas was passed at a controlled flow rate of 150 mL min^−1^. Three to five replicates (100 μL) for each sample were automatically injected into a combustion glass tube filled with a catalyst (Pt-coated Al) at 720 °C in a furnace. The organic matter present in the sample was oxidized on the catalyst to CO_2_. The evolved CO_2_ was measured by non-dispersive infrared detection (NDIR) by integrating peak area.

In order to determine DOC concentrations with high accuracy, it is necessary to reduce the system blank as low as possible. The blank run was continued until organic-free distilled water (DIW) was stable within the detection limit (<5 μM) of our DOC method. Six-point calibration curves for acetanilide were used daily for DOC standardization. The reliability of the measurements was verified on a daily basis by comparing the measured values with a DOC-certified seawater sample (DSR: 44–46 μM for DOC, University of Miami) and procedural blanks. Most of our DSR measurement results (n = 34, 92%) were in good agreement with the DSR values (within 2%), although three measured values differed by 3–5% from the DSR values. These DSR measurements were performed for every 10 samples. The average standard deviation value for all DSR measurements was ±1.2 μM, which is similar to the value (±1 μM) suggested by Carlson *et al*. (ref. 25). An inter-laboratory comparison between the two laboratories involved (Kim and Dittmar) confirmed the accuracy of measurements to be within 5%.

The stable isotope compositions of dissolved organic carbon (δ^13^C-DOC) were measured by using TOC-IR-MS (Isoprime IR-MS coupled with vario TOC cube), which is commercially available (Isoprime, Elementar). This instrument uses the common high temperature catalytic combustion method, which is the same principle as our DOC method. Briefly, 10 mL of filtered samples, following acidification to pH 2 with 6 M HCL, is placed into an auto-sampler. The instrument is programmed to purge the sample for 20–30 min with O_2_ gas to completely remove dissolved inorganic carbon species. Then, 1.5 mL of the sample is injected into Pt-impregnated silica spheres in a quartz tube, where DOC is fully converted to CO_2_ at 750 ^°^C. The CO_2_ gas flows through a halogen trap and a water trap. After detecting DOC concentrations using an NDIR detector, CO_2_ gas enters the interface by O_2_ carrier gas. In the interface, a series of valves are set so that the flow from the TOC and the flow toward the IR-MS system can be separated. The CO_2_ gas, carried by O_2_ carrier gas, is trapped by CO_2_ column in the interface. After switching the valves, the trapped gas is released to IR-MS by helium carrier gas. After passing through a reduction furnace to remove any interfering species, including residual oxygen, CO_2_ gas is transferred to the IR-MS. The δ^13^C content of the CO_2_ is measured using Isoprime IR-MS.

The ion source parameters were fine-tuned every day, and the variations of the reference gas were checked to maintain good stability (standard deviation: <0.01%). The blank was determined by running low carbon water (University of Miami), containing a DOC level lower than 2 μM before the analysis of each sample batch. Certified IAEA-CH6 sucrose (International Atomic Energy Agency, −10.45 ± 0.03%) dissolved in the low carbon water was used for standardization. For every 10 samples, one standard sample was measured to check the stability of measurements. The method used for blank determination and corrections followed the previous method^35^,^36^. Our measurement result of δ^13^C-DOC for DSR was –21.5 ± 0.1%, which agree well with previous results reported by Lang *et al*. (ref. 37) (−21.7%) and by Panetta *et al*. (ref. 35) (−21.4 ± 0.3%). The samples were measured three times, and the values were reproducible within 0.3%.

At all the stations, dissolved oxygen (DO), potential temperature, and salinity were measured using a rosette system with a mounted CTD (SBE 911+). All DO data were from a CTD–DO sensor that was calibrated by the data from grab samples (measured on board using the Winkler titration method). AOU, determined from the same bottle as DOC concentration, was obtained by subtracting the measured DO concentration from the saturated DO concentration which is dependent on temperature and salinity. The temperature sensor used was a SBE–3/F thermometer (resolution 0.0003 ^°^C). The probes were calibrated at Sea-Bird Electronics, USA, prior to each cruise. Reported error margins were standard errors. Wherever appropriate, error propagation was applied.

## Additional Information

**How to cite this article**: Kim, T.-H. *et al*. Extraordinary slow degradation of dissolved organic carbon (DOC) in a cold marginal sea. *Sci. Rep*. **5**, 13808; doi: 10.1038/srep13808 (2015).
